# Evolving Evidence in Idiopathic Intracranial Hypertension

**DOI:** 10.3390/life11111225

**Published:** 2021-11-12

**Authors:** Susan P. Mollan, Heather E. Moss, Steffen Hamann

**Affiliations:** 1 Birmingham Neuro-Ophthalmology, University Hospitals Birmingham NHS Foundation Trust, Birmingham B15 2TH, UK; 2 Department of Ophthalmology, Stanford University, Stanford, CA 94305, USA; hemoss@stanford.edu; 3 Department of Neurology & Neurological Sciences, Stanford University, Stanford, CA 94305, USA; 4 Department of Ophthalmology, Rigshospitalet, University of Copenhagen, 1165 København, Denmark; steffen.hamann@regionh.dk

Idiopathic intracranial hypertension (IIH) is an enigmatic disorder characterized by raised intracranial pressure (ICP) with no known cause and it affects both children and adults [1]. There is a rising incidence of the condition and it more typically affects women of childbearing age [2,3]. Headache is the predominant symptom, and many report migraine-like headaches [4,5]. Due to the nature of the disease, 7% have severe visual loss at presentation caused by papilledema, requiring neurosurgical intervention [2,6]. The complexity of the spectrum of this disorder includes people who have raised ICP, but never develop papilledema [7] and pre-puberty the disease shares some clinical characteristics but is expectantly distinct from the adult phenotype [8]. This Special Issue “Idiopathic Intracranial Hypertension” published in *Life* (ISSN 2075-1729) has highlighted the world-wide research from a multi-professional perspective within the neurosciences. With an increasing number of people living with the disease [2], specialists are gaining new insights into the optimal way to investigate papilledema, diagnose IIH, and determine the best outcomes for care and management.

Diagnosing and managing IIH can be challenging [2] and selected articles underlined the importance of the multidisciplinary team in joint pathways where specialists worked together to provide the best care for patients [1,6,8]. A number of articles investigated methods to aide diagnostic certainty and enable stratification of the disease [9,10,11,12]. An exploratory study used a handheld device to measure the photopic flash electroretinogram and record the photopic negative response. They found that the device was feasible with 84% demonstrating a reliable trace. They also found that they could stratify patients based on severity with the amplitude of the phototopic negative response being significantly smaller in those with more severe disease [9].

Pediatric IIH can be particularly challenging in pre-pubertal minors. Gilbert et al. analysed a case-control study utilizing MRI findings to build a framework for risk stratification for the diagnosis of pediatric IIH. Whilst prospective validation of their model is required, this analysis defined a cut-off point for the perioptic nerve sheath diameter (>5.2 mm) which, when measured according to the technique outlined, had a high sensitivity and specificity for detection of papilledema in pediatric IIH [10]. Mehr et al. also chose to investigate the MRI features that are characteristic in IIH. They used a biomechanical model to demonstrate globe flattening and found that this phenomenon was dependent on a number of factors, including the mechanical properties of the sclera. As globe flattening develops there is an increased observed optic nerve sheath diameter. Ultimately, they found that the magnitude of ICP elevation is the predominant factor in determining the extent of globe flattening [11].

Optical coherence tomography (OCT) imaging of the optic nerve head is the corner stone for longitudinal monitoring of papilledema. Optic disc drusen, and peripapillary hyperreflective ovoid mass-like structures (PHOMS) can make it challenging to interpret quantitative indices alone as they can cause significant elevation of the optic nerve head. Wibroe et al. used a standardized protocol to image the optic nerve head and found that optic disc drusen were no more commonly found, as compared to rates seen in the general population. Importantly nearly one third demonstrated hyperreflective lines, which the authors propose as a consequence of optic disc crowding, with four fifths having PHOMS by three months with no statistical difference in the retinal nerve fibre layer global thickness or in the macular ganglion cell volume compared to patients without PHOMS [12].

In adults with IIH there are well known co-morbidities such as obesity [1,3], mental health diagnoses [1], polycystic ovarian syndrome [13] and obstructive sleep apnoea [14]. In this issue Jensen et al. highlighted a novel observation where 3 non-obese boys diagnosed with IIH also had a diagnosis of autism spectrum disorder [15]. This is worthy of further validation. Those that manage autism spectrum disorder should be aware of this finding and be cognisant of this new potential association to prevent under-diagnosis, and when faced with unexplained vision loss, esotropia, or new onset headaches they should consider IIH within their differential diagnosis [15].

Defining and characterizing the intracranial pressure abnormality in IIH is important. With the ability to measure pulse amplitude, Dr Eide monitored and analysed overnight ICP prior to shunt surgery in IIH patients who were refractory to medical management. He found that the mean wave amplitude was abnormal despite a normalized mean ICP. As he suggested these recordings were taken in people with established disease and this may reflect reduced brain compliance, with a proposed alteration in the glia–neurovascular interface that may impair the astrocytic pulsation absorber mechanisms [16]. Further work in this area is important, as it could help define those in whom surgery is successful, predict revision, or indeed those that remain “shunt” dependent.

Management remains a challenge with no class one evidence for surgery for sight threatening IIH. A number of groups tackled this topic and provided their insights into the various surgical techniques deployed currently for IIH. These included neurosurgical shunting [2,6], optic nerve sheath fenestration [2,17], and venous sinus stenting [2,18]. The most common surgery for sight threatening IIH both in the United Kingdom [2] and USA [19] is neurosurgical shunting and Sunderland et al. provided a detailed dialogue of the choices for devices and a stepwise approach for successful cerebrospinal fluid diversion [6]. They also provided unique data on IIH participants from the BASICS trial which evaluated the efficacy of antibiotic impregnated catheters against standard and silver impregnated catheters establishing superiority of antibiotic catheters in reducing infection rate [20]. Here they showed an increased 2 year revision rate for those with IIH, as compared to those who underwent cerebrospinal fluid diversion for other conditions. While the reasons behind this are not known, overall it appears that revision rate for neurosurgery shunt is falling in the UK [2]. Despite this good news, when comparing the surgical disciplines the 30 day readmission rate was highest for shunting as compared to bariatric surgery, stenting and optic nerve sheath fenestration in IIH [2]. Hagen et al. evaluated the impact of unilateral superomedial transconjunctival optic nerve sheath fenestration [17]. In this retrospective consecutive medically refractory cohort, they noted that higher lumbar puncture opening pressure was predictive of retinal ganglion cell loss. They also were able to determine that surgical delay impacted upon the visual field global outcome of mean deviation [17]. This is important knowledge for clinicians who may have to enlist the help of an expert surgeon, who may not yet understand the priority for surgery when dealing with sight threatening disease in IIH. Venous sinus stenting in the management of IIH has become a popular discussion point and Townsend and Fargen provided an eloquent review supporting its use, and they concluded that carefully crafted prospective controlled studies are an unmet need [18].

Disease remission from weight management remains a medical and social challenge in many healthcare systems. The IIH:Weight Trial was the first to compare the efficacy and cost-effectiveness of bariatric surgery with a dietary intervention in the setting of a randomised control trial [21,22]. The cost-effectiveness of bariatric surgery improved over time and therefore the incremental cost of surgery when offset against the incremental reduction of ICP improved after 24 months [22]. In order to change healthcare policy, it is not only clinical benefit but cost efficacy that needs to be provided [21,22,23].

In conclusion, this Special Issue has advanced the field of IIH. It has provided novel insights into how the disease can be stratified, it has carefully unpicked the patient pathways that exist across specialties and importantly analysed how we treat sight threatening disease. The expert authors collected here have a wealth of knowledge working in this rare condition and know that the field needs to propel itself forward to further understand the pathophysiology, validate its models and above all improve the path for all patients from disease to remission.

## Data Availability

Not applicable.
